# Influence of host origin on host choice of the parasitoid *Dinarmus basalis* : Does upbringing influence choices later in life?

**DOI:** 10.1093/jis/14.1.26

**Published:** 2014-01-01

**Authors:** F. Sankara, L. C. B. Dabiré, Z. Ilboudo, S. Dugravot, A. M. Cortesero, A. Sanon

**Affiliations:** 1 Laboratoire d’Entomologie Fondamentale et Appliquée, UFR/SVT, Université de Ouagadougou, 03 BP 7021 Ouagadougou 03, Burkina Faso; 2 Laboratoire d’Entomologie Agricole de Kamboinsé, Institut de l’Environnement et de Recherches Agricoles (INERA), 01 BP 476 Ouagadougou 01, Burkina Faso; 3 UMR 1349, Institut de Génétique, Environnement et Protection des Plantes (IGEPP), Université de Rennes1, 263 av du Général Leclerc, 35042 Rennes Cedex, France

**Keywords:** adaptation, behavior, behaviour, complex secondary hosts, host location, integrated control

## Abstract

The aim of this study was to investigate the influence of volatile compounds from four secondary host plants on the ability of *Dinarmus basalis* Rond. (Hymenoptera: Pteromalidae) to locate, recognize, and parasitize its host, 4 ^th^ instar larvae or pupae of *Callosobruchus maculatus* F. (Coleoptera: Chrysomelidae). To examine this, strains of *D. basalis* were transferred from cow-pea seeds ( *Vigna unguiculata* (L.) Walp. (Fabales: Fabaceae)) to pigeon pea ( *Cajanus cajan* (L.) Millsp.) and two varieties of Bambara groundnut ( *Vigna subterranea* (L.) Verdc.) seeds. The ability of *D. basalis* females to recognize the volatile compounds emanating from their complex host plant was tested by using a Y-tube olfactometer and a three-dimensional device. The results suggest that when females have a choice between pure air and the air emanating from their com-plex host of origin, they are attracted to the air tainted by the volatile compounds they have become accustomed to. They spent significantly more time ( *p* < 0.0001) in the branch of the tube leading to the odorous air than in the tube leading to the pure air. When females from pigeon pea seed hosts were offered a choice between cowpea and pigeon pea seeds, all containing 4 ^th^ instar larvae, the familiar odor of pigeon pea seeds were most attractive. When females from Bambara groundnut (white and striped) seed hosts were offered a choice between cowpea and pigeon pea seeds, all containing 4 ^th^ instar larvae, they were significantly attracted to the odour of cowpea seeds. In the three-dimensional system, the females from the four strains did not appear to have any preference for a given type of seed containing 4 ^th^ instar larvae or pupae. The parasitism rate remained high on all four types of seeds used. These results show that the use of *D. basalis* as a biological control agent is possible in host changing situations where *C. maculatus* starts to attack other legumes. The results of this study also provide information supporting the behavioral plas-ticity of *D. basalis* . Understanding the mechanisms involved in the adaptive phenomena of biological control agents is discussed in the context of the development of adequate methods of pest control.

## Introduction

*Dinarmus basalis* Rond. (Hymenoptera: Pteromalidae) is a solitary ectoparasitoid of larvae and pupae in the family Bruchidae. These hosts, which can be found within seeds of the bean family Fabaceae, provide both a laying site and a food stock for the developing offspring. *D. basalis* females lay their eggs on the host tegument within the seed. After hatching, the *D. basalis* larvae will attach to their Bruchid host and develop ( Sanon et al. 1998 ; Sanon et al. 2005 ). The postembryonic development of this species involves five instars ( Caubet 1993 ).

Studies using *D. basalis* as a biological control against legume-eating Bruchids were carried out in South Asia ( Islam and Kabir 1995 ), Latin America ( Schmale et al. 2002 ; Schmale et al. 2006 ), and West Africa ( Mondedji et al. 2002 ; Sanon et al. 2005 ; Amevoin et al. 2007 ). However, studies focusing on the ability of this parasitoid to locate its host within unusual host plant seeds are scarce.

Parasitoids use a wide range of physical and chemical cues during the search for their hosts ( Godfray 1994 ; Kumazaki et al. 2000 ; Seagraves 2009 ). A sequence of responses to different information brings the wasp closer and closer to its potential host ( Gauthier et al. 2002 ; Conord 2006 ; Seagraves 2009 ). There are three levels of chemical information. chemical cues perceptible at long, medium, and short distances. There are also contact chemicals detected when the wasp touches the host substrate and/or the host itself ( Kennedy 1977 ; Vinson 1991 ).

The volatile compounds of plants involved in the host-search behavior of *D. basalis* at long distances become increasingly important as the parasitoid approaches the oviposition site ( Godfray 1994 ; Hoffmeister and Roitberg 1997 ). The success of the parasitoid in the host search depends on its response to the chemical cues coming from the host and the host plant ( Gandolfi et al. 2003 ; Seagraves 2009 ).

The biology and behavior of these parasitoids are influenced by the plant seeds in which their hosts develop (Cortesero 1994; Gu and Dorn 2000 ; Gandolfi et al. 2003 ; Seagraves 2009 ). There are a wide range of stimuli associated with a parasitized host that are able to induce a change in the behavior of conspecific or allospecific female parasitoids. Several categories have been defined based on the nature of the stimulus involved ( Godfray 1994 ; Nufio and Papaji 2001 ), the location of the stimulus on the host plant system ( Salt 1937 ; Hofsvang 1990 ), and the primary function of the stimulus ( Seeley 1998 ; Nufio and Papaji 2001 ).

The location of the stimulus determines the phase of the host-search behavior, where discrimination is made ( Jaloux et al. 2004 ; Jaloux et al. 2005 ). One theory is that the recognition of the host is due to the perception of the female parasitoid that the stimulus is situated on the surface of the seed. This is thought to be related to the exploitation of the host by *D. basalis* . Parasitoids invest a lot of time locating a suitable host ( Hoffmeister and Roitberg 1997 ). In the host-searching process, there is a direct link between the search for a suitable host and reproductive success ( Gauthier et al. 2002 ).

Because the larvae (and/or pupae) of *Callosobruchus maculatus* Fabricius (Coleoptera: Chrysomelidae) live inside the seeds of host plants, host discrimination by the parasitoid *D. basalis* cannot be studied directly. Therefore, female behavior was studied indirectly through response to odors released by seed complexes (seeds containing 4 ^th^ instar larvae or pupae of *C. maculatus* ).

The aim of this study was to determine the influence of volatile substances emitted from four types of seed complexes on the ability of *D. basalis* to locate, recognize, and parasitize its host.

## Materials and Methods

### Parasitoid rearing

Seeds containing *C. maculatus* 4 ^th^ instar larvae or nymphs were infested by adult male and female *D. basalis* for 48 hours. The *D. basalis* were then removed, and the seeds containing larvae or pupae, whether or not they had been parasitized, were raised until adult emergence of the insects. These *D. basalis* adults were isolated and used for experiments or for the maintenance of the strain by regular rearing. This rearing method has been described by several authors ( Sanon et al. 1998 ).

### Procedure for obtaining four strains of *Dinarmus basalis*

The four strains were obtained by transferring *D. basalis* adults from cowpea seeds ( *Vigna unguiculata* (L.) Walp. (Fabales: Fabaceae) to seed complexes from pigeon pea ( *Cajanus cajan* (L.) Millsp.), other cowpea seeds, and white and striped varieties of Bambara groundnut ( *Vigna subterranea* (L.) Verdc.) plants. After emergence, adult parasitoids were isolated and maintained through the same process of rearing described above on each of the four varieties of legumes. This operating process was started three years before the experiments were conducted.

### Influence of strain origin on host location in a Y-tube olfactometer in four strains of *Dinarmus basalis*

Experiments were conducted in a Y-tube olfactometer comprising one proximal branch (1) and two distal arms or test arms (2 and 3). The proximal branch is the common part of the tube. The proximal branch and the distal arms were each 25 cm in length. The distal arms were each connected to their own cylindrical container, which was used as an odorous chamber. Both containers had the same dimensions (height = 13 cm and diameter = 9 cm). These chambers were connected to a flow meter for measuring airflow from a humidifying chamber (glass bottle containing distilled water). Nitrogen-oxygen gas was passed through the humidifying chamber from a 25 kg bottle. The Y-tube was placed in a climate-and light-controlled room at 30 ± 2° C and 450 Lux.

The experiments were carried out using 1–2-day-old naïve *D. basalis* females without oviposition experience from the four strains. These females were isolated from the seeds as they emerged. The test aimed to place individual females from a given strain in the proximal branch of the tube and to follow their movement toward the two arms receiving the test odours for three min.

Thirty females of each strain were tested. After each test, the tube was rinsed out with clean air. When five females were tested, the connections between the cylindrical containers, the source of the odor, and the edges of the test arms of the tube were interchanged. At the end of each series of tests, the odorous rooms were washed with soapy water and rinsed with alcohol at 70° C in order to avoid contamination.

Two sets of experiments were performed according to the odors tested. In the first series of tests, *D. basalis* females had a choice between pure air and odorous air emanating from seed complexes of their host plants of origin. In this case, the arm distributing odorous air was connected to a cylindrical container containing 100 g of cowpea seeds, pigeon pea seeds, or either of the two varieties of Bambara groundnut. In the second series of tests, *D. basalis* females of the four strains had the choice between the odour emanating from cowpea seed complexes and the odour from the complex secondary hosts. In this case, one of the odorous chambers contained 100 g of cowpea seed complexes and the other contained 100 g of pigeon pea or either white or striped Bambara groundnut seed complexes.

For all experiments, a stopwatch was used to measure time. It was triggered as soon as the insect began to move within the common branch of the Y-tube. The movement into an area was validated when the insect had passed its entire thorax (which warrants its antennae bathed well) into the visited area ( Conord 2006 ). For each female, the time spent in the distal arms (2 and 3) of the Y-tube was measured.

### Influence of strain origin on host location in a 3D device in four strains of *Dinarmus basalis*

This experiment was conducted in a free choice situation. *D. basalis* females of the four strains had the choice between cowpea seeds, white and striped Bambara groundnut, and pigeon pea seeds, all containing 4 ^th^ instar larvae or pupae. A 3D device was used.

The 3D device consisted of a central rectangular box (10 x 8 x 4.5 cm) with four identical peripheral boxes located at equal distances (8 cm) on each side. In each peripheral box, cowpea, white or striped Bambara groundnut, or pigeon pea seeds were introduced, each contributing to a total of 25 *C. maculatus* 4 ^th^ instar larvae.

Finally, *D. basalis* females were isolated and grouped into four batches from the cowpea (A), white Bambara groundnut (B), striped Bambara groundnut (C), and pigeon pea (D) strains. Each batch consisted of 400 mature females (1–2 days old). Twenty females of the same strain were introduced into the central box to observe their behavior in the device every six hours for a total of 24 hours. At each observation period, the distribution of insects in the different boxes of the device was recorded. After 24 hours, the insects were removed and the infested seeds were transferred into Petri dishes, which were maintained at rearing conditions (32 ± 0.1° C, 36 ± 1% RH) and observed until adult emergence. Twenty replications were carried out for each strain.

The mean number of insects on each type of seed was calculated. Emerged parasitoids as well as bruchids were counted and parasitism rate (Rp) was estimated by calculating the ratio between the total number of parasitoids emerged and the total number of parasitoids and bruchids that emerged. It was determined by the following formula ( Sanon et al. 1998 ):

$Rp = \frac{Np}{Np+Nb}\times 100$

Where Np = total number of parasitoids emerged, and Nb = total number of bruchids emerged.

### Statistical analysis

Data were analysed using SAS version 8 (SAS, www.sas.com ). The time spent in the two arms of the Y-tube was expressed as mean values obtained for the 30 tested females. These data were submitted to ANOVA.

Separation of means was done using a *t* -test for paired series to compare differences between means. The values of parasitism rate are presented as mean ± SE of the 20 recorded values. They were then submitted to oneway ANOVA followed by a Fisher LSD test at a significance level of 5%.

## Results

### Influence of strain origin on host location in a Y-tube olfactometer in four strains of *Dinarmus basalis*

**Figure 1.**
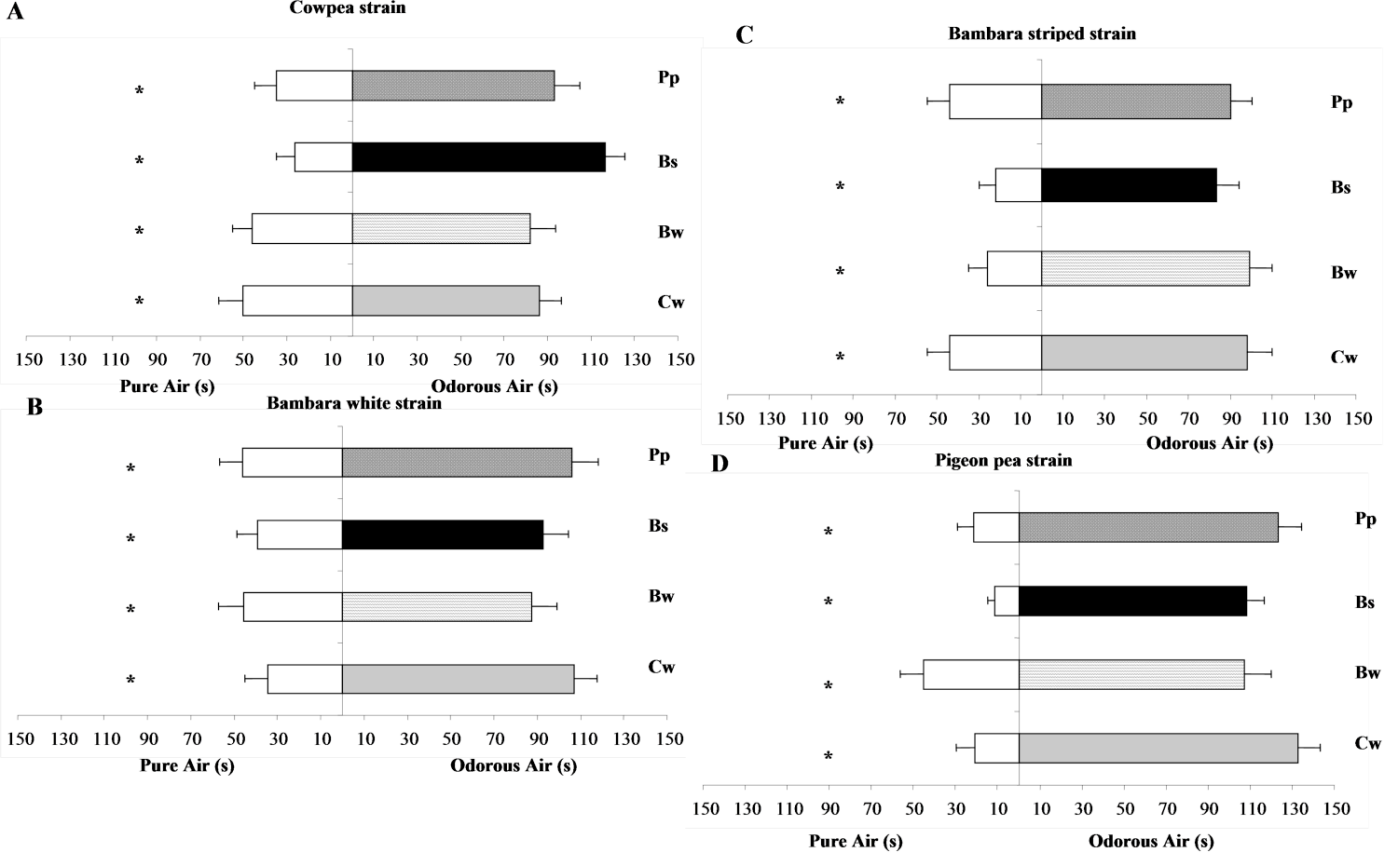
Response (time in seconds in each branch) of naïve females of four strains of *Dinarmus basalis* in a Y-tube olfactometer disseminating pure air and odorous air. Means ± SE differed significantly according to the *t* -test of paired series at the significance level of 5%. Cw = odor of cowpea seeds ( *Vigna unguiculata* ); Bw = odor of white Bambara groundnut seeds ( *Vigna subterranea* ); Bs = odor of striped Bambara groundnut seeds ( *V. subterranea* ); Pp = odor of pigeon pea seeds ( *Cajanus cajan* ). High quality figures are available online.

When *D. basalis* females from all four strains had a choice between pure air and air containing odors from seeds containing 4 ^th^ instar lar-larvae or pupae of their plants of origin, they were attracted to the odorous air, where they spent significantly more time ( *p* < 0.01) ( Figure 1 ).

**Figure 2.**
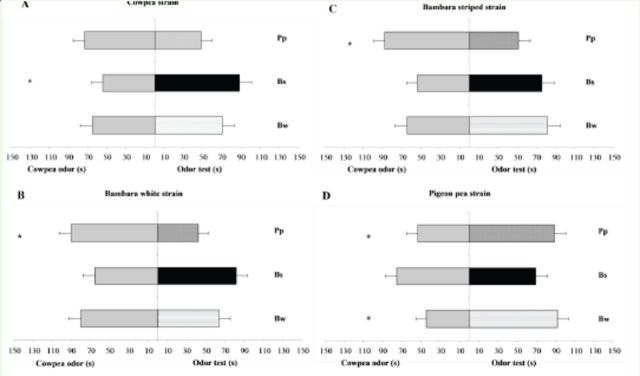
Response (time spent in seconds in each branch) of naïve females of four strains of *Dinarmus basalis* in a Y-tube olfactometer disseminating the odour of cowpea seeds ( *Vigna unguiculata* ) containing 4th instar larvae or pupae and the odors of each of the other plants (pigeon pea ( *Cajanus cajan* ) and white and striped Bambara groundnut ( *Vigna subterranea* )) containing 4th instar larvae or pupae. Means ± SE differed significantly according to the *t* -test of paired series at the significance level of 5%. Cw = odor of cowpea seeds; Bw = odor of white Bambara groundnut seeds; Bs = odor of striped Bambara groundnut seeds; Pp = odor of pigeon pea seeds. High quality figures are available online.

When *D. basalis* females had the choice between the odor of cowpea seeds and the odor of seeds of the other plants, their responses varied relative to their strain of origin. Females reared on cowpea seeds chose the odor of striped Bambara groundnut seeds over the odor of cowpea seeds, where they spent significantly more time ( *p* = 0.04) ( Figure 2A ).

Females from the two varieties of Bambara groundnut seeds chose the odor of cowpea seeds over the odor of pigeon pea seeds. They spent significantly more time in contact with the odor emanating from the cowpea than with the odour coming from pigeon pea ( *p* = 0.003 with white Bambara odour; *p* = 0.03 with striped Bambara odour; Figure 2B , C ).

Finally, female offspring of pigeon pea seeds were significantly attracted to the odor of white Bambara groundnut seeds and pigeon pea seeds rather than the odor of cowpea seeds ( *p* = 0.037; Figure 2D ).

### Influence of strain origin on host location in a 3D device in four strains of *Dinarmus basalis*

**Figure 3.**
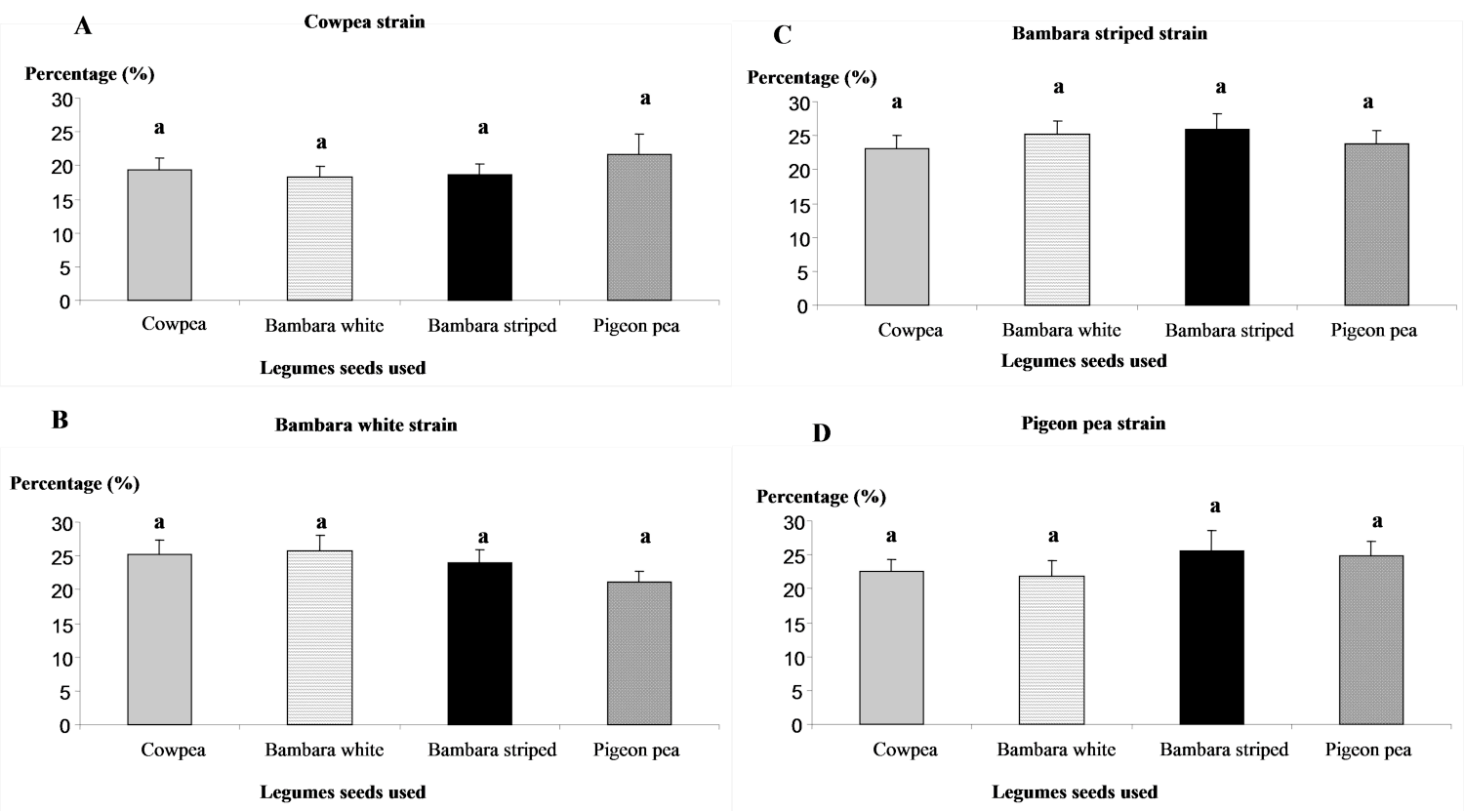
Distribution (means ± SE) of female *Dinarmus basalis* in a situation of free choice in a 3D device between cowpea ( *Vigna unguiculata* ), white and striped Bambara groundnut ( *Vigna subterranea* ), and pigeon pea ( *Cajanus cajan* )). Treatments with the same letter are not statistically different according to the Fisher LSD test at a significance level of 5%. High quality figures are available online.

In a free choice situation in the 3D system, females from all the strains did not have a clear preference for a given type of seed. These females were invariably distributed after 24 hours in the four boxes containing the seeds of legume hosts ( *p* > 0.05) ( Figure 3 ).

### Parasitism rate

The parasitism rate observed in females raised on the cowpea strain was high and almost identical on the seeds of all plants ( Table 1 ).

**Table 1.**
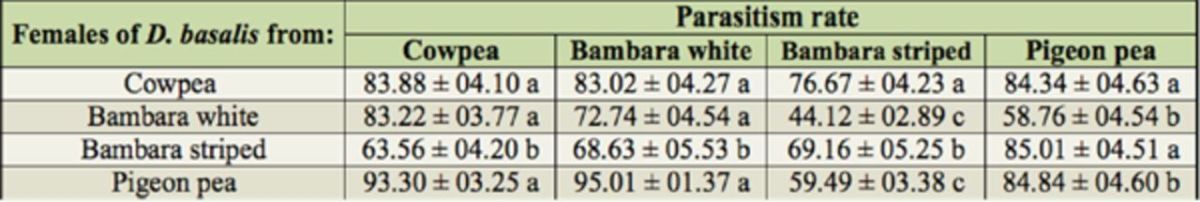
Variation of parasitism rate in four strains of *Dinarmus basalis* .

Means ± SE are compared in the lines; values followed by different alphabetic letters are significantly different at *p* ≤ 0.05 using the Fisher LSD test.

Females raised on white Bambara groundnut seeds exhibited significantly higher parasitism rates on cowpea seeds than on pigeon peas and striped Bambara groundnuts ( *p* < 0.0001 with pigeon pea; *p* < 0.0001 with striped Bambara; Table 1 ).

Females raised on striped Bambara groundnut seeds had high rates of parasitism on pigeon pea seeds, which differed significantly from those raised with other seed species ( *p* = 0.0031 with cowpea; *p* = 0.0216 with white Bambara; *p* < 0.026 with striped Bambara; Table 1 ).

Females raised on pigeon pea seeds had high rates of parasitism on cowpea seeds ( Table 1 ). These results differed significantly from those obtained from females raised on pigeon pea and striped Bambara groundnut seeds ( *p* =0.03 with pigeon pea; *p* < 0.0001 with striped Bambara).

## Discussion

*D. basalis* females were able to recognize complex host odors and move towards the source of these odors. They were able to locate their hosts using olfactory stimuli, even when they were lodged in unfamiliar substrates. These results support those of Sanon et al. (2006) . According to these authors, when naïve *D. basalis* females have a choice between pure air and odors from seed complexes, they are more attracted to the odorous air. It is also well-known that the learning mechanism during upbringing plays an important role in host location by the parasitoid wasp.

These learning capacities represent an important mechanism underlying behavioral plasticity in parasitoids ( Vet and Dicke 1992 ; Takasu and Lewis 2003 ). Learning capacities have been observed in *Eupelmus vuilleti,* a parasitoid species sympatric to *D. basalis* . Host-location behavior depends on the prominent chemical environment at the beginning of the parasitoid’s life (Cortesero and Monge 1994; Monge and Cortesero 1996 ). Some studies have also shown that learning of olfactory cues occurs mainly during adult emergence or in young adults ( Turlings et al. 1993 ; Du et al. 1997 ; Fujiwara et al. 2000 ). According to Sanon et al. (2006) , these parasitoids were able to learn a novel odor associated with their host plant complex during their development or during an oviposition-probing phase. Therefore, the success of host location depends on both internal and external cues from the whole environment in which the wasp has developed.

For entire the host-searching and location processes to occur successfully, a hierarchy of behavioral stimuli and associated responses with the host and its habitat takes place ( Turlings et al. 1993 ). A parasitoid wasp detects chemical cues associated with the host, as well as those associated with host plant, and has the capacity to integrate and respond to different external and internal information during the host-searching process ( Desouhant et al. 2005 ). In *D. basalis* , internal cues, specifically those within the pupal chamber on the host surface, involve direct handling of the host before its quality can be assessed. Internal stimuli are of low accessibility, but are the most reliable indicators of previous parasitism ( Gauthier et al. 2002 ). External cues at the host site or habitat could be more quickly detected than internal ones ( Hoffmeister and Roitberg 1997 ; Gauthier et al. 2002 ). Volatile cues resulting from herbivore activity make the seeds more attractive than healthy ones and therefore are used by *D. basalis* to locate potential oviposition sites.

The preference of *D. basalis* females for seed odors compared to pure air could be explained by the fact that the prolonged development of *D. basalis* within seed complexes increased their sensitivity to the complexes’ odors. The sensitivity of female parasitoid wasps to the odors emitted by their hosts is not innate, but induced at the end of larval development ( Monge and Cortesero 1996 ; Gandolfi et al. 2003 ).

Once emitted, the odors of seed complexes may attract females and stimulate their loco-motive activity ( Monge and Cortesero 1996 ). The result of this attraction could lead to the location of the host, immigration to the host ( Seagraves 2009 ), and host recognition, all of which were observed in this study.

The preference for cowpea seed compared to pigeon pea seed odors of female offspring originating from Bambara groundnut could be explained by the chemical environment in which the parasitoid developed ( Corbet 1985 ; Gandolfi et al. 2003 ; Seagraves 2009 ). Host selection in parasitic wasps requires the use of a wide range of chemical signals from not only the host but also from its substrate ( Lewis and Martin 1990 ; Tumlinson et al. 1993 ; Gandolfi et al. 2003 ; Seagraves 2009 ). Cowpea and Bambara groundnut are species belonging to the same genus and therefore may have similar or identical volatile compounds. Thus, females raised on Bambara groundnut seeds that have acquired sensitivity to Bambara groundnut odors during development may be similarly attracted to the volatiles of cowpea seeds.

Furthermore, *D. basalis* raised on white and striped Bambara groundnut seeds may also be more attracted to the odor of cowpea than that of pigeon pea, which is an unfamiliar complex host. Similarly, females raised on pigeon pea seeds appear to have a preference for pigeon pea seeds, which may again be explained by a specific sensitivity, acquired during their development within the seeds, to volatiles of this species ( Gandolfi et al. 2003 ). Upon emergence, females of some parasitic wasps have a specific sensitivity to odors emanating from the seeds of the species they were reared from (Cortesero 1994; Gandolfi et al. 2003 ; Jaloux et al. 2005 ; Seagraves 2009 ).

In the 3D device, *D. basalis* was able to recognize and locate its host even within an unfamiliar substrate, showing that parasitoids use some stimuli to locate their host successfully ( Gauthier et al. 2002 ; Nicole 2002 ). These stimuli may be visual or olfactory and perceptible at least at short distances ( Jaloux et al. 2005 ; Conord 2006 ; Seagraves 2009 ). They are attractive and appear to trigger the oviposition behavior in *D. basalis* ( Jaloux et al. 2004 ). In the 3D device, *D. basalis,* in addition to using olfactory senses, could see the seeds located in the peripheral boxes and thus used vision in addition to smell to locate its host ( Seagraves 2009 ).

Regardless of the complex host species it was raised on, *D. basalis* females migrated to the different types of seeds equally in the 3D device. A possible explanation for this would be to find sufficient food for the survival of its offspring. Females may be sensitive to the quantity of available hosts in the various pe-ripherals boxes during their migration, which may explain their distribution. According to Seagraves (2009) , the abundance and quality of hosts in a habitat affect the reproductive activity of a female in that habitat. Thus, areas with more hosts would attract more females.

A second hypothesis is that there is no difference in sensitivity among females of *D. basalis* raised from the four different complex host plant strains, especially in the perception of stimuli emitted at a short distance by seeds from different species.

Several factors could explain the high rate of parasitism seen in *D. basalis* females that were raised on the four plant species. It may be due to climatic conditions (temperature and relative humidity) or host availability. All experiments were carried out under a mean temperature of 32 ± 0–1° C, which, according to some authors, was among the temperatures that allowed optimal development of *C. maculatus* and *D. basalis* ( Sanon et al. 1998 ; Mondedji et al. 2002 ; Amevoin et al. 2007 ). Considering the high variation in parasitism rates among different the strains, it can be inferred that there is a difference in sensitivity to stimuli between females raised on different plant species. This difference is due to differences in the chemicals on the seed coat, which induce oviposition in *D. basalis* females ( Jaloux et al. 2004 ). The low parasitism rate observed on striped Bambara groundnut seeds compared to parasitism rates on the seeds of other species may be explained by a high mortality during larval development. It may also be explained by differential abilities of *D. basalis* females to perforate the tegument of the seeds in order to deposit eggs on the *C. maculatus* 4 ^th^ instar larvae, which varies with plant host species. Low parasitism on Bambara groundnut seeds may also be explained by *D. basalis* females not appreciating the taste of the seeds.

*D. basalis* recognizes its hosts even when they are lodged in substrates other than cowpea seeds. Females are able to locate their hosts at short distances using complex olfactory and visual stimuli and at longer distances through the use of olfactory stimuli. *D. basalis* can complete its developmental cycle with varying degrees of success. In this study, the host plants that *D. basalis* females originated from had an influence on the selection of a new host for oviposition. These results show that the use of *D. basalis* as a biological control agent is possible in host changing situations when *C. maculatus* attacks other legumes. This study also provides information supporting the behavioral plasticity of *D. basalis* . Understanding the mechanisms involved in the adaptive phenomena of biological control agents is crucial in developing adequate methods of pest control.
